# Hepatic microRNA-126 deficiency restrains liver regeneration through p53 pathway in mice

**DOI:** 10.1038/s41392-020-00395-1

**Published:** 2021-01-28

**Authors:** Lingling Zhang, Yugang Qiu, Fan Yang, Jiyuan Yao, Ying Wang, Yang Qin, Hanchuan Mou, Qing Jing, Leiming Liu, Zhenyu Ju

**Affiliations:** 1 Key Laboratory of Aging and Cancer Biology of Zhejiang Province, Institute of Aging Research, School of Medicine, Hangzhou Normal University, Hangzhou, China; 2 School of Rehabilitation Medicine, Weifang Medical University, Weifang, China; 3 School of Public Health and Management, Weifang Medical University, Weifang, China; 4 Key Laboratory of Regenerative Medicine of Ministry of Education, Regenerative Medicine and Health Guangdong Laboratory, Institute of Aging and Regenerative Medicine, Jinan University, Guangzhou, China; 5 CAS Key Lab of Tissue Microenvironment and Tumor, Shanghai Institute of Nutrition and Health, Chinese Academy of Sciences, Shanghai, China

**Keywords:** Reproductive disorders, Metabolic disorders

**Dear Editor,**

Emerging evidences show close associations between miRNAs and liver regeneration. MiR-126 is known as a governor of vascular integrity and angiogenesis. MiR-126 is involved in the self-renewal of hematopoietic stem cells (HSCs) and leukemia stem cells.^1,2^ MiR-126 also plays a vital role in hepatocellular carcinogenesis,^3^ however, the functions of miR-126 in liver regeneration were still unclear.

To explore hepatic functions of miR-126, we developed miR-126 liver specific knockout (LKO) mice (Supplementary Fig. 1a). MiR-126 was almost undetectable in the hepatocytes of miR-126 LKO mice (Supplementary Fig. 1b, c, Supplementary Fig. 2a). The body weights showed no differences between two groups (Supplementary Fig. 2b). The liver to body weights and hepatic morphologies were also comparable between two groups (Supplementary Fig. 2c, d). The liver injury related index, alanine aminotransferase (ALT), aspartate aminotransferase (AST), albumin (ALB), total protein (TP), and total bile acids (TBA) levels in miR-126 LKO mice were all comparable to control group (Supplementary Fig. 3a–c). The levels of TG, low-density lipoprotein (LDL-c), and high-density lipoprotein (HDL-c), free fatty acid (FFA) also showed no differences (Supplementary Fig. 3d). The glucose level, glucose and insulin tolerance tests showed no significant differences (Supplementary Fig. 4a–c). To further elucidate the role of hepatic miR-126 in glucose and TG regulation, we fed control and miR-126 LKO mice with high fat diet (HFD). Hematoxylin and eosin (H&E) staining showed that the lipid droplets accumulated in both control and miR-126 LKO mice livers after HFD fed (Supplementary Fig. 5a). The glucose tolerance and insulin sensitivity in miR-126 LKO mice were comparable to control HFD group (Supplementary Fig. 5b, c). Plasma AST, ALT, ALB, TP, and TBA levels showed no significant differences between control and miR-126 LKO mice after HFD fed (Supplementary Fig. 6a, c). There were also no differences in the levels of TG, HDL-c, LDL-c, FFA between control and miR-126 LKO mice after HFD fed (Supplementary Fig. 6d). These results indicated that hepatic miR-126 deficiency had no obvious effect on glucose and TG levels in plasma.

To elucidate role of miR-126 during liver regeneration, we examined the expression of miR-126 in the perfused hepatocytes following sham or partial hepatectomy (PH) at 0, 24, 36, 48, and 72 h in C57BL/6 mice. The miR-126 expression peaked at 36 h after PH (Fig. 1a). Next, we performed two-thirds partial hepatectomy in control and miR-126 LKO mice. The liver to body weight significantly decreased during liver regeneration (Supplementary Fig 7a). The peak of hepatocyte proliferation in miR-126 LKO mice was significantly lower compared with control livers following PH, as indicated by BrdU and Ki67 incorporation assay in vivo, Ki67 and EDU staining in vitro (Fig. 1b–d and Supplementary Fig 7b, c). Accordingly, the expression levels of proliferating cell nuclear antigen (PCNA) were significantly decreased at 36 and 48 h after PH compared with the corresponding control mice (Fig. 1e–g). Taken together, the data indicated that deletion of miR-126 hindered hepatocytes proliferation and miR-126 was essential for liver regeneration.

**Fig. 1:**
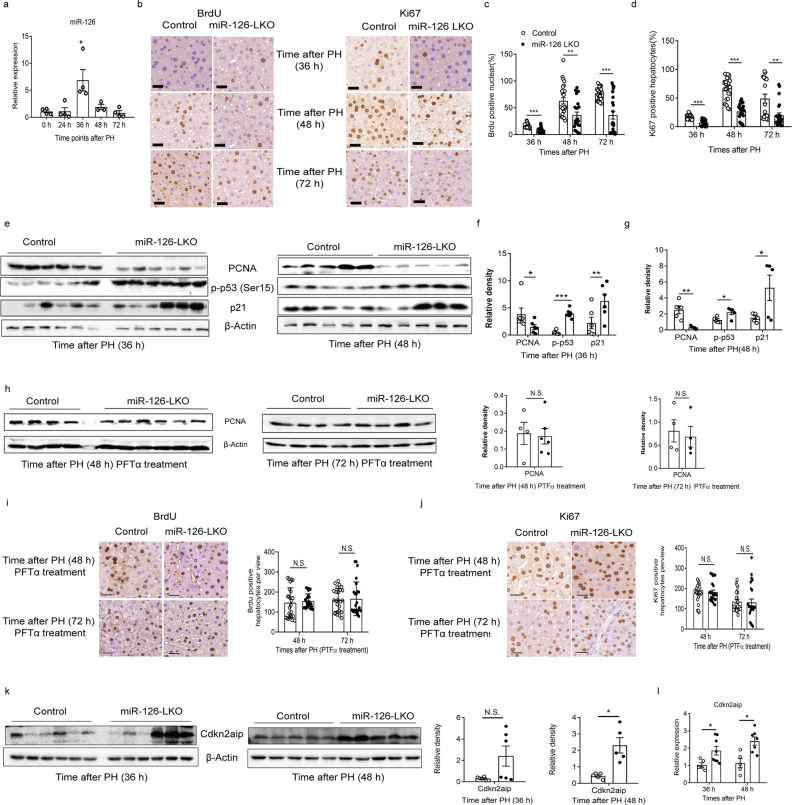
MiR-126 is essential for liver regeneration through inhibiting the Cdkn2aip-p53 pathway. **a** Relative expression of miR-126 in the perfusd hepatoctes before and after PH at different time points. Quantifications were normalized to U6. (0, 24h, 36h, 48h, and 72h). **b** Representative IHC staining of BrdU and Ki67 positive cells in Control and miR-126 LKO livers at 36h, 48h, and 72h following PH. Scale bar: 50 µm. **c**, **d** The quantification figures of the IHC staining of BrdU and Ki67 in control and miR-126 LKO livers at different time points (36h, 48h, and 72h) after PH. Five sections per mouse were examined, five mice per group. **e** Representative western blotting results of PCNA, p-p53, p21 in livers at different time points (36h, 48h) after PH. **f**, **g** The quantification figure of the western blotting analysis of PCNA, p-p53, p21 expression of livers in control and miR-126 LKO group at different time points (36 h, 48 h) after PH. Quantifications were normalized to β-actin (*n* ≥ 5 per group). **h** Representative and quantification figures of western blotting results of PCNA expression in PFT-α treated control and miR-126 LKO livers at 48 h, 72 h following PH. Quantifications were normalized to β-actin (*n* ≥ 4 per group). **i**, **j** Representative quantification figures of IHC staining of BrdU and Ki67 positive cells in PFT-α treated control and miR-126 LKO livers at 48h and 72h following PH. Scale bar: 50 µm. Five sections per mouse were examined, five mice per group. **k** Representative and quantification figures of western blotting results of Cdkn2aip in livers at different time points (36h, 48h) after PH. Quantifications were normalized to β-actin (*n* ≥ 5 per group). **l** RT-PCR analysis of cdkn2aip expression in control and miR-126 LKO mice 36h after PH. Quantifications were normalized to β-actin (*n* ≥ 5). Data are presented as mean±SEM. *P < 0.05; **P < 0.01; ***P < 0.001, Wilcoxon test in (**k**), student’s test in (**a**, **c**, **d**, **f**, **g**, **h**, **j**, **l**). PH partial hepatectomy, h hour, PCNAproliferating cell nuclear antigen, LKO liver specific knockout, N.S. no significant difference

MiR-126 regulates HSCs cycle and maintains HSCs quiescence through PI3K-AKT signaling pathway.^1^ We observed dramatic expression of p-AKT (473) and p27 in the livers of miR-126 LKO and control mice after PH. However, the difference between two groups was not significant (Supplementary Fig. 8a–c). Forced miR-126 expression in mouse and human progenitors B cells reduced p53 transcriptional activity.^2^ Following PH, the levels of phospho-p53 and p21 were significantly increased at 36 h, 48 h after PH in miR-126 LKO livers (Fig. 1e–g; Supplementary Fig. 8d, e). These findings indicated that p53-p21 pathway might be responsible for the decreased liver regeneration in miR-126 LKO mice. After p53 inhibitor pifithrin-α (PTF-α) treatment, the expression of p-p53 and p21 were comparable between two groups after PH (Supplementary Fig. 9a, b). PTF-α treatment almost abolished the difference in PCNA expression between two groups at 48, 72 h post-PH (Fig. 1h). PTF-α treatment also consistently eliminated the differences in BrdU and Ki67 staining between two groups at 48 h and 72 h post-PH (Fig. 1i, j). The effect of PTF-α induced p53 inhibition on liver regeneration after PH was controversial in different reported model.^4,5^ However, the activity of p53 in miR-126 LKO mice was significantly higher than control group. The upregulated activation of p53 pathway induced by miR-126 deficiency was contributed to the inhibition of liver regeneration. Over-expression of miR-126 in the hematopoietic system directly downregulates Cdkn2aip,^2^ which is a positive regulator of p53 activity. In line with this, both the mRNA and protein levels of Cdkn2aip mice were significantly increased in miR-126 LKO mice compared to control mice at 36 and 48 h after PH (Fig. 1k, l). The activity of p53-p21 pathway was also significantly downregulated after the knockdown of Cdkn2aip in isolated primary hepatocytes (Supplementary Fig. 9c). Taken together, these results validated during liver regeneration, miR-126 regulated p53 signaling by targeting Cdkn2aip (Supplementary Fig. 9d).

In summary, we firstly used the hepatic conditional knockout mice to elucidate the role of miR-126 in liver and liver regeneration. Our study showed specific deletion of miR-126 in the liver decreased the rate of liver regeneration after PH. Deficient liver regeneration in miR-126 LKO mice was a result of the activation of p53 pathway. Furthermore, deletion of miR-126 increased the expression of Cdkn2aip, which is a positive regulator of p53 activity. Disruption of p53 pathway rescued liver regeneration in miR-126-deficient mice. Therefore, miR-126 could be beneficial to liver regeneration in mice through modulating the activity of p53. The present study elucidated the role of miR-126 in liver and liver regeneration by genetic depletion, had a therapeutic potential for acute liver failure, cirrhosis, or small-for-size liver transplantations in human.

## Supplementary information

supplementary file

Supple Fig 1a-c

Supple Fig 2a-d

Supple Fig 3a-d

Supple Fig 4a-c

Supple Fig 5a-c

Supple Fig 6a-d

Supple Fig 7a-c

Supple Fig 8a-e

Supple Fig 9a-d
