# Supplementary material for: Hepatic microRNA-126 deficiency restrains liver regeneration through p53 pathway in mice
Source: Signal Transduct Target Ther. 2021 Jan 28;6:32. doi: 10.1038/s41392-020-00395-1 (PMC7841169; doi:10.1038/s41392-020-00395-1)
Supplement: Supplementary file 1 — supplementary file [file 41392_2020_395_MOESM1_ESM.pdf]

Supplemental Material for

Hepatic microRNA-126 Deficiency Restrains Liver Regeneration Through p53 Pathway In Mice

This supplementary data file contains:

- **Supplementary Figure legend 1-9**
- **Supplementary Table 1-2**
- **Supplementary Methods**
- **References**

Supplementary Fig.1 Construction of the miR-126 LKO mice strategy.

(a) The structure of knocking out miR-126 strategy in liver.
(b) Relative expression of miR-126-5P in the hepatocytes of control and miR-126 LKO mice. Quantifications were normalized to U6 (n=3).
(c) Relative expression of miR-126-3P in the hepatocytes of control and miR-126 LKO mice. Quantifications were normalized to U6 (n=3).
Data are presented as mean ± SEM. ***P<0.001. Abbreviations: LKO, liver specific knock out.

Supplementary Fig.2 Normal histological morphology in miR-126 LKO mice.

(a) Relative expression of pre-miR-126 in the hepatocytes of control and miR-126 LKO mice, Quantifications were normalized to U6 (n=8).

(b) Body weight in the control (n⩾7) and miR-126 LKO (n⩾8) mice.

(c) Liver to body weight in the normal condition of control (n=8) and miR-126 LKO (n=7) mice.
(d) Representative HE staining of the liver in the control and miR-126 LKO group. Scale bar: 20 μm.
Data are presented as mean ± SEM. ***P<0.001. Abbreviations: HE, hematoxylin-eosin; LKO, liver specific knock out; N.S., no significant difference.

Supplementary Fig. 3 Normal biochemical index in miR-126 LKO mice.

(a) The levels of AST (U/L), ALT (U/L) in the serum of control and miR-126 LKO group (n≥11).
(b) The levels ALB (g/L), TP (g/L) in the serum of control and miR-126 LKO group (n≥11).
(c) The leves of TBA (μmol/L) in the serum of control and miR-126 LKO group (n≥11).
(d) The levels of TG (mmol/L), HDL-c (mmol/L), LDL-c (mmol/L), FFA (mmol/L) in serum of control and miR-126 LKO group (n≥11).
Data are presented as mean ± SEM. *P<0.05; **P<0.01; ***P<0.001. Abbreviations: LKO, liver specific knock out; ALT, alanine aminotransferase; AST, aspartate aminotransferase; ALB, albumin; TP, total protein; TBA, total bile acid; FFA, free fatty acid; GLU, glucose; TG, triglyceride; HDL-c, high-density lipoprotein cholesterol; LDL-c, light-density lipoprotein cholesterol.

Supplementary Fig.4 Normal plasma glucose level in miR-126 LKO mice.
(a) The glucose level in the serum of control and miR-126 LKO mice under AL and fasted condition (n≥8).
(b) The ITT test results in control and miR-126 LKO mice (n≥5 at each time point).
(c) The GTT test results in control and miR-126 LKO mice (n=6 at each time point).

Data are presented as mean ± SEM. Abbreviations: LKO, liver specific knock out; AL, ad libitum feeding; ITT, insulin tolerance test; GTT, glucose tolerance test; min, minites; N.S., no significant difference.

Supplementary Fig.5 Normal plasma glucose level in miR-126 LKO mice after HFD treatment.

(a) Representative HE staining of the liver in control and miR-126 LKO group under the HFD condition.

(b) The ITT test results in control and miR-126 LKO mice under the HFD condition. (n=3 at each time point).

(c) The GTT test results in control and miR-126 LKO mice under the HFD condition. (n=3 at each time point).

Data are presented as mean ± SEM. Abbreviations: LKO, liver specific knock out; AL, ad libitum feeding; ITT, insulin tolerance test; GTT, glucose tolerance test; min, minites; N.S., no significant difference.

Supplementary Fig.6 Normal biochemical index in miR-126 LKO mice after HFD treatment.

(a) The levels of AST (U/L), ALT (U/L) in the serum of control and miR-126 LKO group under the HFD condition (n=3).

(b) The leves of TBA (μmol/L) in the serum of control and miR-126 LKO group under the HFD condition (n=3).

(c) The levels ALB (g/L), TP (g/L) in the serum of control and miR-126 LKO group under the HFD condition (n=3).

(d) The levels of TG (mmol/L), HDL-c (mmol/L), LDL-c (mmol/L), FFA (mmol/L) in serum of control and miR-126 LKO group under the HFD condition (n=3).

Data are presented as mean ± SEM. Abbreviations: HFD, high fat diet; LKO, liver specific knock out; ALT, alanine aminotransferase; AST, aspartate aminotransferase; ALB, albumin; TP, total protein; FFA, free fatty acid; GLU, glucose; TG, triglyceride; HDL-c, high-density lipoprotein cholesterol; LDL-c, light-density lipoprotein cholesterol.

Supplementary Fig.7 The hepacocytes proliferation was decreased in miR-126 LKO mice.

(a) The liver to body weight in control and miR-126 LKO mice during liver regeneration (n⩾4 at each time point).

(b) The proportion of ki67 positive cells in the isolated hepatocytes in control and miR-126 LKO mice after 2 hours culture in vitro (n=3).

(c) The proportion of EDU positive cells in the isolated hepatocytes in control and miR-126 LKO mice after 4 hours culture in vitro (n=4).

Data are presented as mean ± SEM. Abbreviations: LKO, liver specific knock out;

PH, partial hepatectomy; h, hour; Data are presented as mean ± SEM. *P<0.05, **P<0.01.

Supplementary Fig.8 The deficient liver regeneration in miR-126 LKO mice was dependent on p53 but not AKT pathway.

(a) Western blot results of P-AKT (473) and p27 expression in control and miR-126 LKO mice at 36 and 48h after 2/3 PH. Quantifications were normalized to β-actin (n≥5) at each time point.

(b) Western blot analysis of P-AKT (473) expression in control and miR-126 LKO mice at 36 and 48h after 2/3 PH. Quantifications were normalized to β-actin (n≥5) at each time point.

(c) Western blot analysis of p27 expression in Control and miR-126 LKO mice at 36 and 48h after 2/3 PH. Quantifications were normalized to β-actin (n≥5) at each time point.

(d) Western blot results of p-p53 (ser15) and p21expression in control and miR-126 LKO mice at 72h after 2/3 PH.

(e) Western blot analysis of P-p53 (ser15) and p21expression in Control and miR-126 LKO mice at 72h after 2/3 PH. Quantifications were normalized to β-actin (n≥5) at each time point.

Data are presented as mean ± SEM. *P<0.05. Abbreviations: PH, partial hepatectomy; h, hour; LKO, liver specific knock out; N.S., no significant difference.

Supplementary Fig.9 The p53 expression after PFT-α treatment.

(a) Western blotting results of p53, p21 expression in livers of PFT-α treated control and miR-126 LKO at 48h after PH. Quantifications were normalized to β-actin (n≥4) at each time point.

(b) Western blotting analysis of p53, p21 expression in livers of PFT-α treated control and miR-126 LKO at 48h after PH. Quantifications were normalized to β-actin (n≥4) at each time point.

(c) The activity of p53-p21 pathway was significantly downregulated after the knockdown of Cdkn2aip at 48 hours in isolated primary hepatocytes between two groups.

(d) The illustrator of the work path of miR-126 during liver regeneration.

Data are presented as mean ± SEM. Abbreviations: PH, partial hepatectomy; h, hour; LKO, liver specific knock out; N.S., no significant difference.

Supplemental table 1

Sequences of oligonucleotides used in this study

**Gene**	**Forward primer**	**Reverse primer**
**U6**	5’-CTCGCTTCGGCAGCACATATACT- 3’	5’-ACGCTTCACGAATTTGCGTGTC-3’
**miR-126**	5’-ACTTTTGGTACGCGCTGTGA-3’	5’-CGCGCATTATTACTCACGGT-3’
**miR-126-5p**	5’- CATTATTACTTTTGGTACGCG -3’	
**miR-126-3p**	5’- TCGTACCGTGAGTAATAATGCG-3’	

**Cdkn2aip**	5'- CAGTTGAGCGAGATCATGGGA-3'	5'- TGGCGCTATCCTGATGGGA-3'
**Cdkn2aip-siRNA1**	5'- GGAUAGCGCCACAAGUGAATT-3'	5'- UUCACUUGUGGCGCUAUCCTT-3'
**Cdkn2aip -siRNA2**	5'- GCAGAAGUAGAGUUGCCAUTT-3'	5'- AUGGCAACUCUACUUCUGCTT-3'
**Cdkn2aip -siRNA3**	5'- CCAGUGAGAGUUCUGUCAATT-3'	5'- UUGACAGAACUCUCACUGGTT-3'
**siNC**	5'- UUCUCCGAACGUGUCACGUTT–3'	5'- ACGUGACACGUUCGGAGAATT-3'

Supplementary table 2. antibodies used in Western blotting

**Antibody**	**Dilution**	**Source**
PCNA	**1:2000**	Cell Signaling Technology
p21	**1:500**	Santa Cruz
p27	**1:500**	Santa Cruz
p-p53 (Ser 15)	**1:1000**	Cell Signaling Technology
β-actin	**1:10000**	Sigma
Cdkn2aip	**1:1000**	Sigma
p-AKT (473)	**1:1000**	Cell Signaling Technology

Materials and methods

Animals. The miR-126flox/flox mice were generated in Qing Jing's lab, Shanghai Institute of Nurition and Health, CAS. Briefly, the first loxP was inserted into host gene Egfl7 intron 7-5' end, and the second loxP together with neomycin-resistant gene flanked by FRT sites was inserted into intron 7-3' end. Genetic transmission was confirmed by backcrossing the chimera with C57BL/6J mice and the Neo-cassette was removed using FLPeR mice. The floxed mice used in this study were maintained in C57BL/6J with at least four backcrosses. MiR-126$^{flox/flox}$ mice were crossed with Albumin-Cre (alias Alb-Cre; The Jackson Laboratory) mice to generate liver specific knock out (LKO) mice with the genotype miR-126$^{flox/flox}$-Alb-Cre mice. MiR-126$^{flox/flox}$

mice were used as littermate controls. All the mice were crossed back to C57B6/J ground. As for HFD feeding test, the animals receive HFD (Research Diet, D12492) at 4 weeks old for six weeks. All animals received humane care according to the criteria outlined in the NIH's Guide for the Care and Use of Laboratory Animals, and all animal experiments were approved by the Animal Care and Ethics Committee at Hangzhou Normal University. All the mice used for the experiments are 8-12 weeks old.

PH Experiments. PH was performed using 8- to 14-week-old miR-126 LKO mice and their control littermates (of the C57BL/6 background) according to the method described previously[1]. More than 12 mice from each group were sacrificed at the indicated time point. All mice were injected intraperitoneally with 100mg/kg body weight BRDU (Sigma Chemical Co., St. Louis, MO) two hours before sacrifice. Liver remnants were removed, weighed, and either snap-frozen in liquid nitrogen or processed for histology.

Liver History and Immunohistochemistry. Liver tissues were fixed in 0.01 M phosphate buffered saline (pH7.4) containing 10% formalin and then embedded in paraffin, sectioned, and stained with hematoxylin eosin (HE) for histological examination. Immunohistochemistry was performed using mouse anti-BRDU (Sigma Chemical Co., St. Louis, MO), and anti-Ki67 (CST, Mouse mAb).

Q-PCR Experiments. MicroRNAs were extracted from liver tissue samples according to the kit instruction (Axygen, AP-MN-MIRNA). Then for miRNA first-strand cDNA synthesis and qRT-PCR to quantify mature miRNA expression for miR-126a-5p, miR-126a-3p, or U6 according to instruction (Mir-X miRNA First-Strand Synthesis and TB Green qRT-PCR, Takara). The primers sequences are listed in supplemental table 1. Total RNA was isolated from liver samples using TRIZOL (Invitrogen, CA) and then reverse-transcribed and analyzed by real-time quantitative PCR with SYBR Green

master mix (Bio-Rad, CA) and target-specific primers (supplemental table 1) on Bio-Rad CFX96 Touch™. The relative levels were calculated using the comparative-Ct method ($2^{-\Delta\Delta}$Ct method).

Immunoblot analysis

Frozen liver tissue samples (50 mg) were homogenized in 1 ml of lysis buffer (beyotime, Shanghai, China) with protease inhibitor cocktail (Roche, Mannheim, Germany). Liver extracts underwent SDS-PAGE electrophoresis at 120 V. They were transferred onto 0.4 μm nitrocellulose membranes. The antibodies against PCNA, p21, p27, phosphorated (p-) p53, β-actin were used as primary antibodies. A VersaDocTM Imaging Systerm was used to visualize proteins and Quanlity one software (Bio-Rad Labrotory Inc, Hercules, CA) was used to analyse the band. Primary Abs are listed in supplemental table 2.

Biochemical analysis

The blood of animal before and after PH were collected. The measure methods were described before[2]. All samples were tested on a HITACHI Clinical Analyzer 7180, according to the manufacturer's protocols. The chemical indexes including TP, TBA, ALB, ALT, AST, TCHOL, HDL, LDL, GLU, TG, FFA. Reagents for serum biochemical analysis were purchased from DiaSys Diagnostic Systems GmbH (Holzheim, Germany).

Primary hepatocytes culture.

Hepatocytes were isolated from Control and miR-126 LKO mice by collagenase perfusion and cultured as previously described[3]. The freshly isolated hepatocytes were cultured for 2h and then analyzed by FACS (LSRFortessa, BD Biosciences). Proliferation was determined by Ki67 (BD Biosciences) Flow Kit and EDU cell proliferation kit (Beyotime Biotechnology). The transient knockdown of Cdkn2aip was

performed in primary hepatocytes isolated from control and miR-126 LKO mice by electroporation according to the protocol (Lonza 4D-Nucleofector™ X Unit, Cat No. V4XP-3024).

Statistical Analysis. Statistical analyses were performed using the Student two-tailed t test and the Wilcoxon test. Differences with calculated P values less than 0.05 were considered to be statistically significant. All data were tested for at least three times. For immunohistochemistry test, we choose about 10 plus views for checking the number of the positive cells. And we use Prism for analyzing the meaning of the data. For western blotting, we use the image pro-plus software to analyze the density of the bands which are gotten from the WB experiments.

References:

1 Mitchell, C. & Willenbring, H. A reproducible and well-tolerated method for 2/3 partial hepatectomy in mice. *Nature Protocol* **3**, 1167-1170, doi:10.1038/nprot.2008.80 (2008).

2 Liu, L. *et al.* Hepatic Tmem30a Deficiency Causes Intrahepatic Cholestasis by Impairing Expression and Localization of Bile Salt Transporters. *The American journal of pathology* **187**, 2775-2787, doi:10.1016/j.ajpath.2017.08.011 (2017).

3 Block, G. D. *et al.* Population expansion, clonal growth, and specific differentiation patterns in primary cultures of hepatocytes induced by HGF/SF, EGF and TGF alpha in a chemically defined (HGM) medium. *Journal of Cell Biology* **132**, 1133-1149 (1996).
