# Supplementary figures and images for: Hepatic microRNA-126 deficiency restrains liver regeneration through p53 pathway in mice
Source: Signal Transduct Target Ther. 2021 Jan 28;6:32. doi: 10.1038/s41392-020-00395-1 (PMC7841169; doi:10.1038/s41392-020-00395-1)

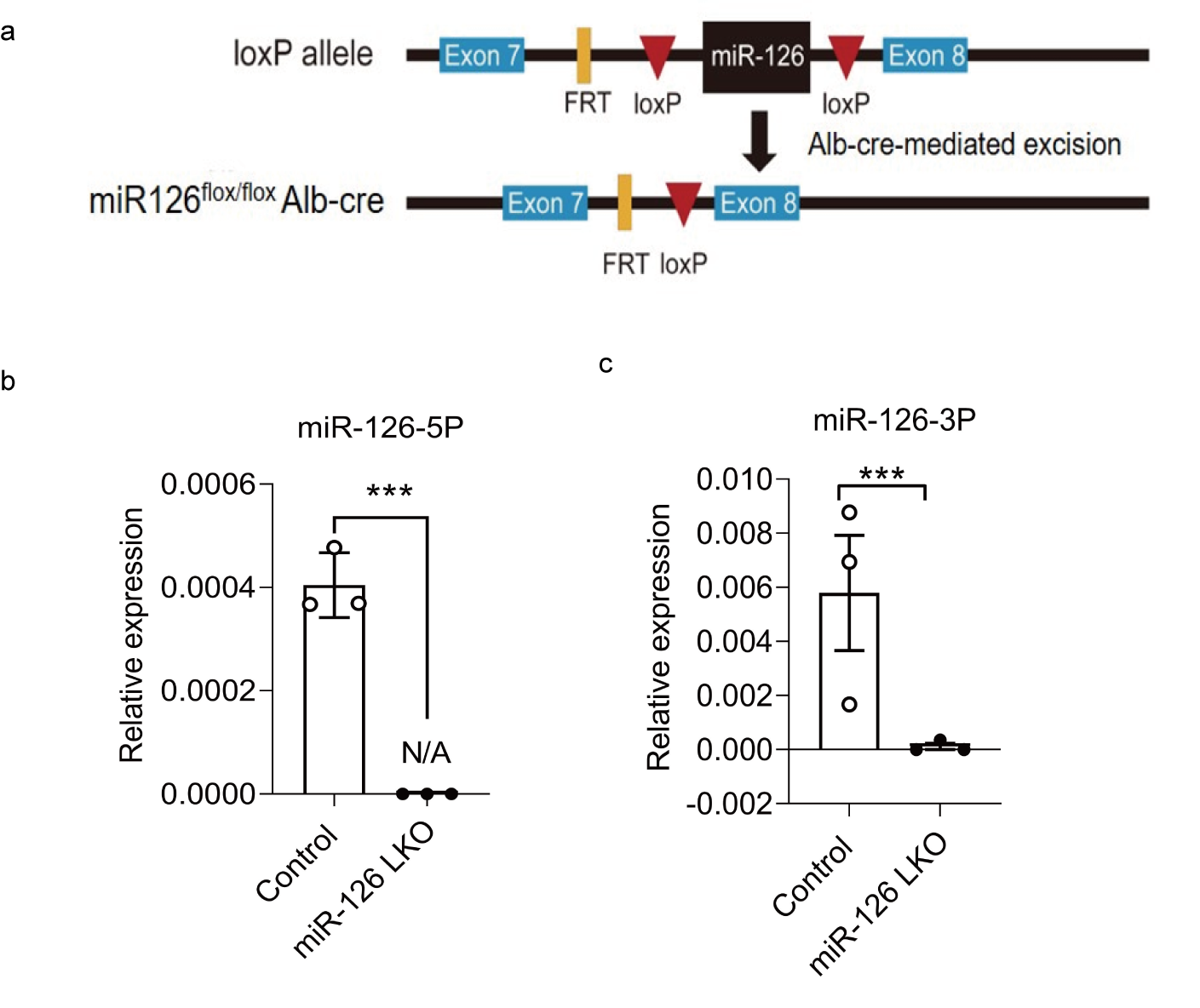

Supplement: Supplementary file 2 — Supple Fig 1a-c [file 41392_2020_395_MOESM2_ESM.tif]

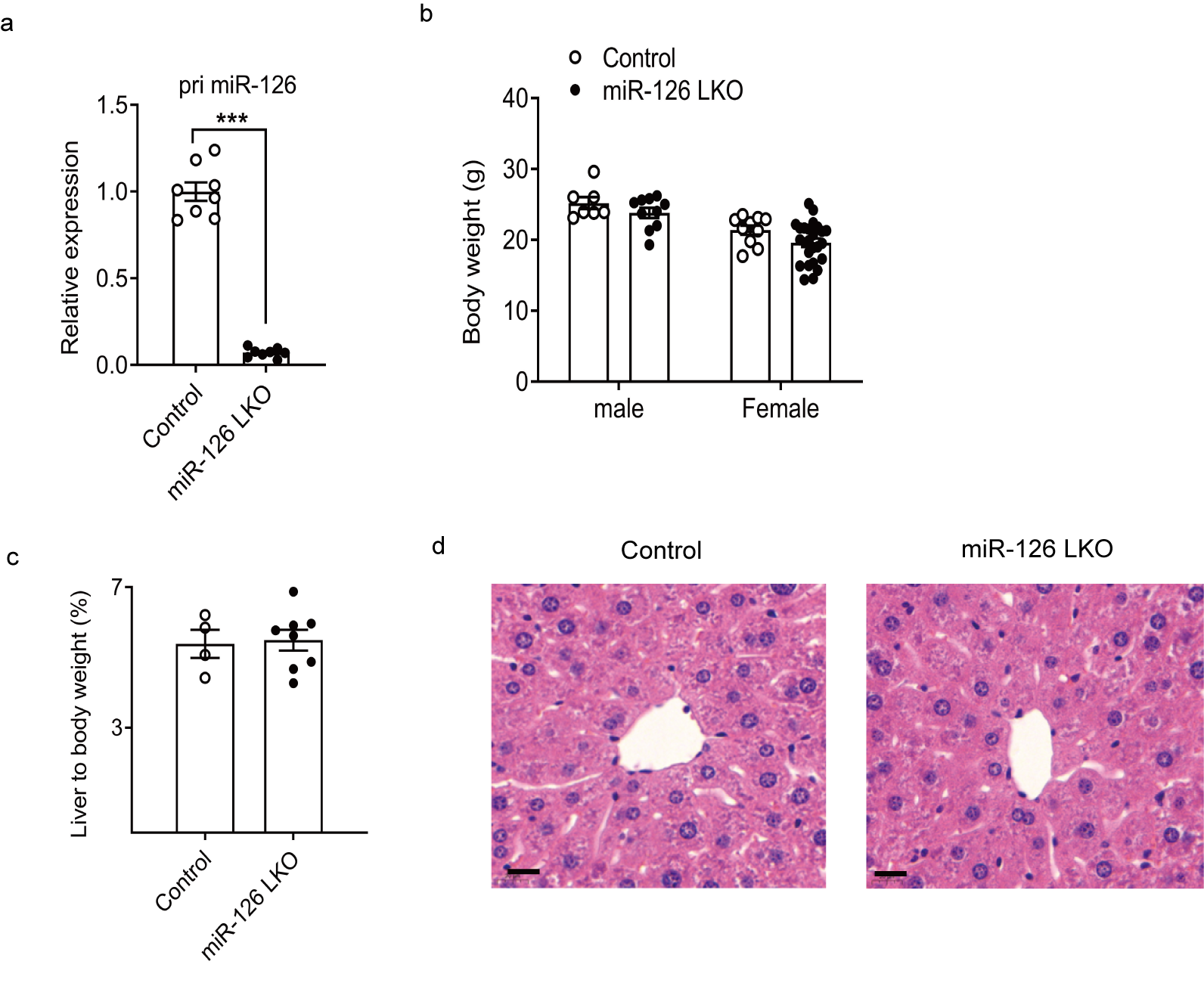

Supplement: Supplementary file 3 — Supple Fig 2a-d [file 41392_2020_395_MOESM3_ESM.tif]

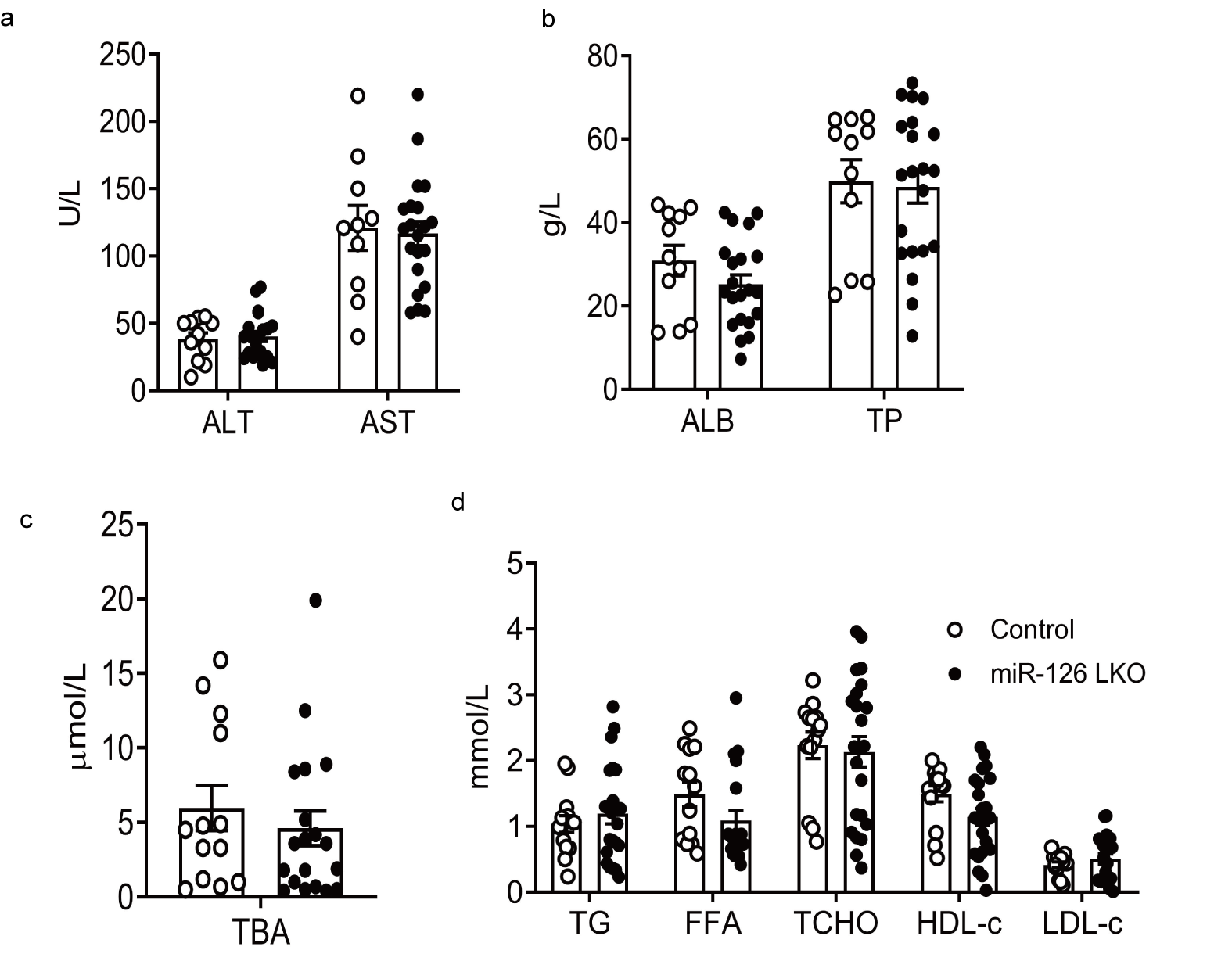

Supplement: Supplementary file 4 — Supple Fig 3a-d [file 41392_2020_395_MOESM4_ESM.tif]

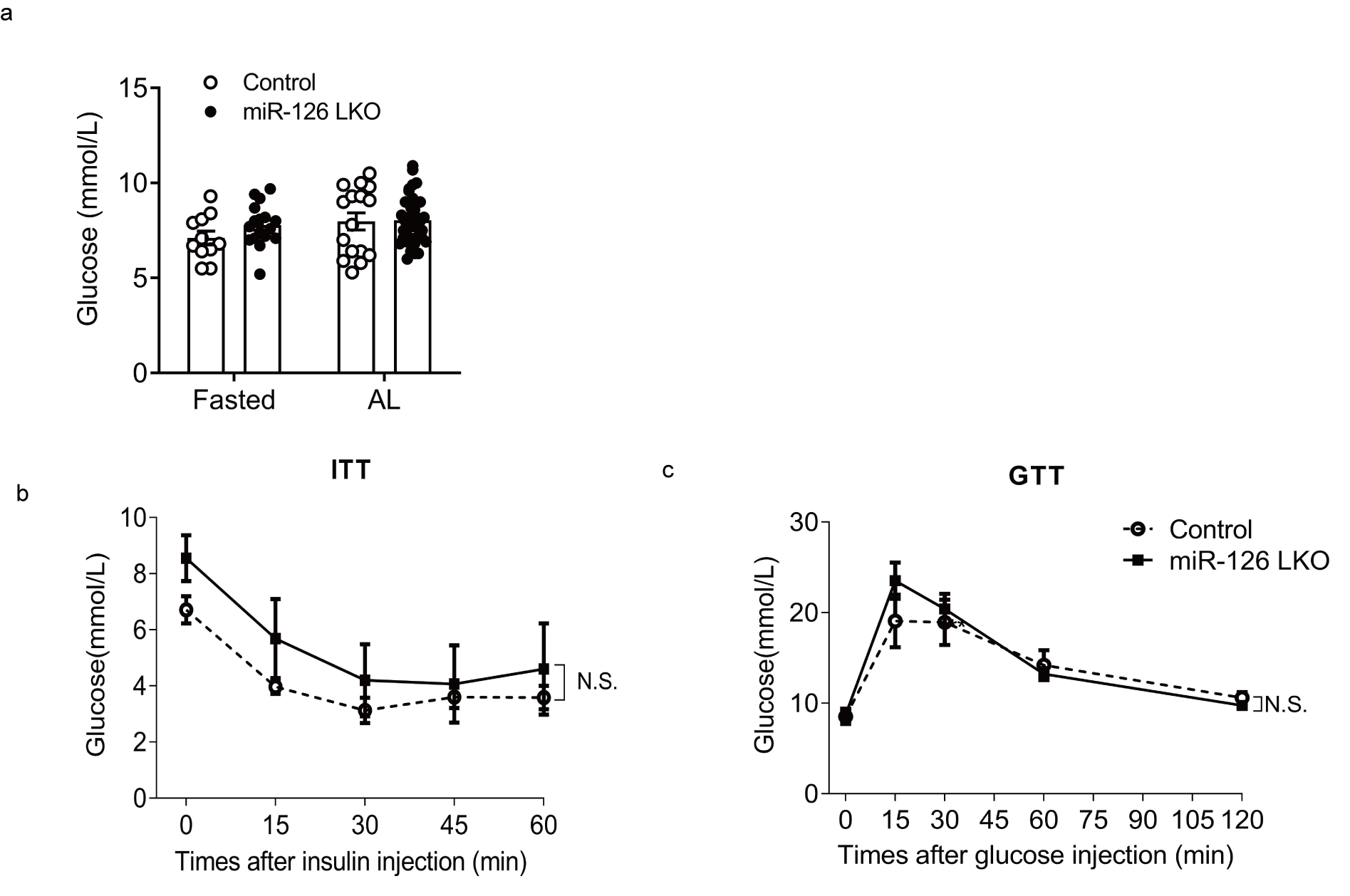

Supplement: Supplementary file 5 — Supple Fig 4a-c [file 41392_2020_395_MOESM5_ESM.tif]

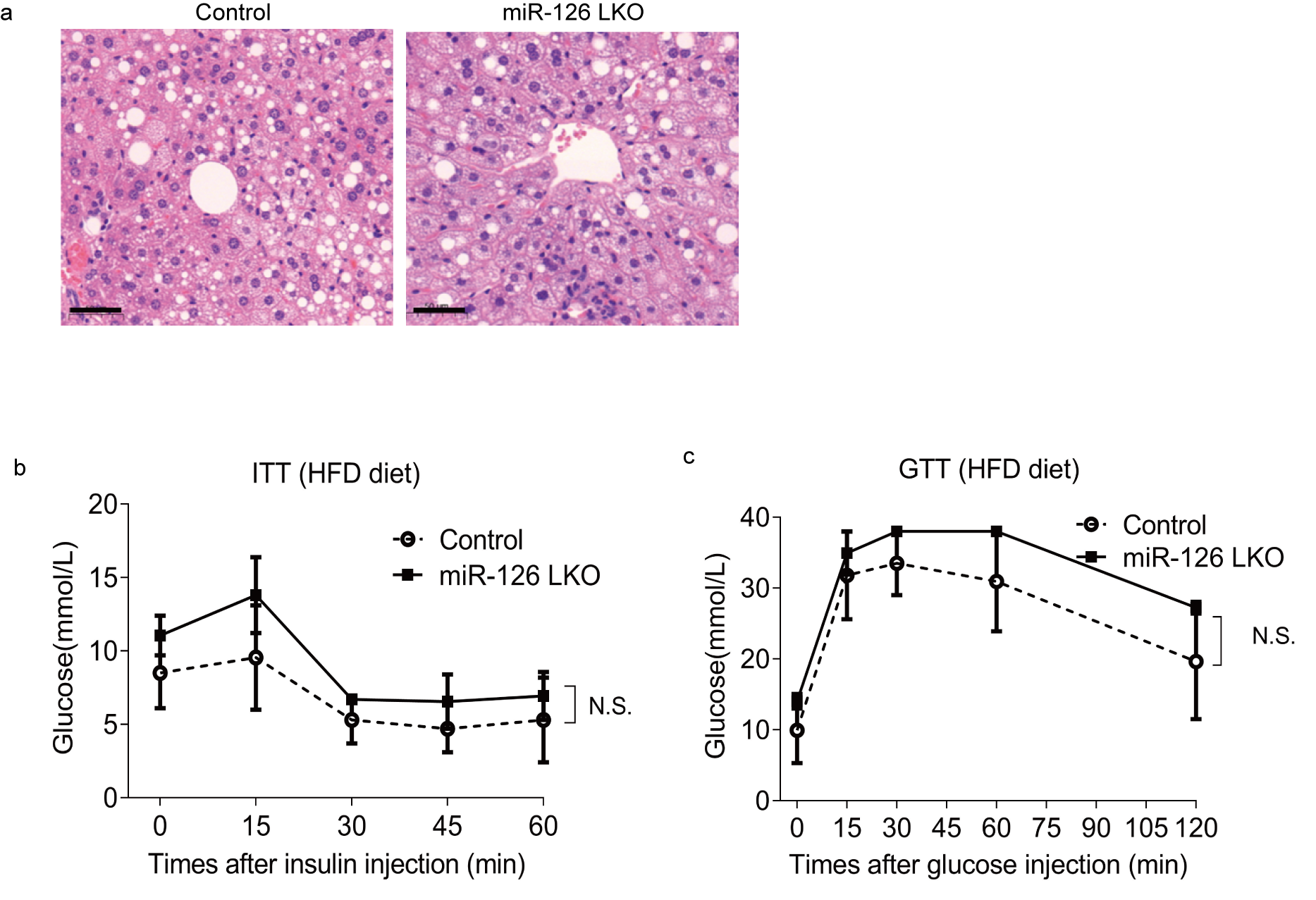

Supplement: Supplementary file 6 — Supple Fig 5a-c [file 41392_2020_395_MOESM6_ESM.tif]

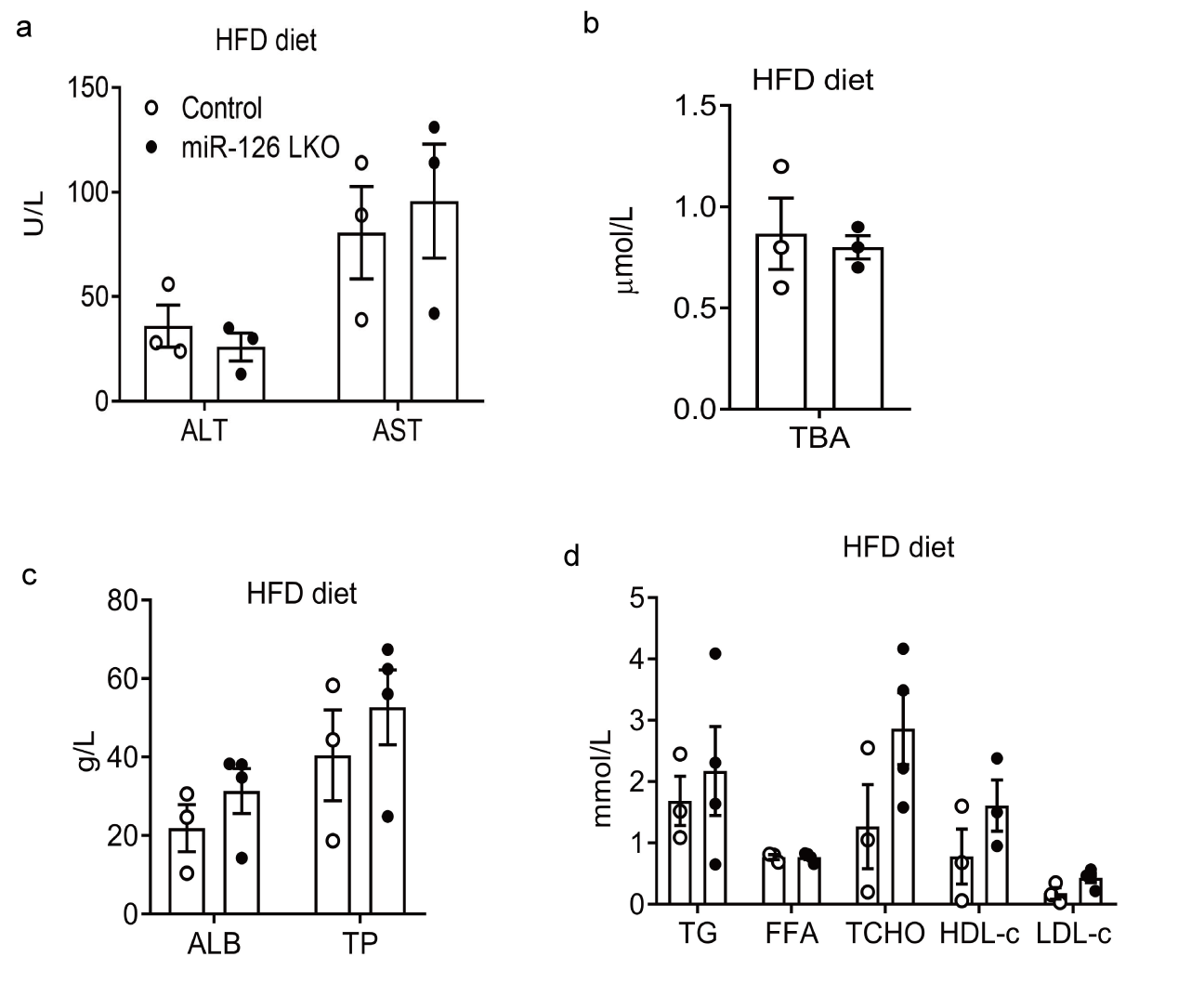

Supplement: Supplementary file 7 — Supple Fig 6a-d [file 41392_2020_395_MOESM7_ESM.tif]

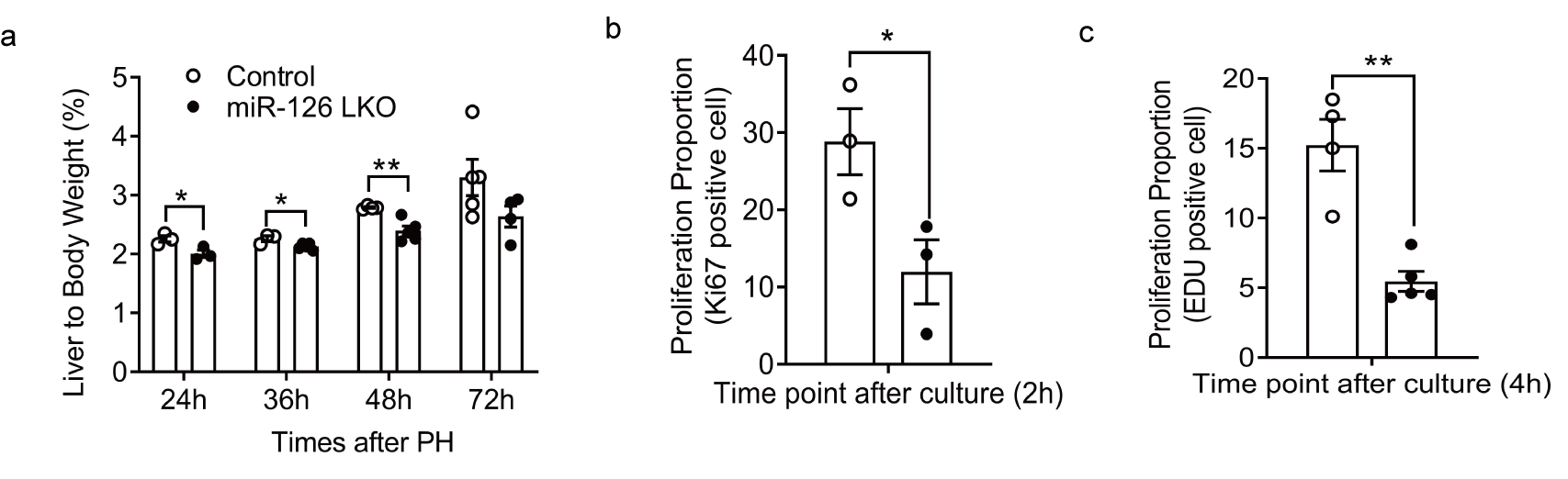

Supplement: Supplementary file 8 — Supple Fig 7a-c [file 41392_2020_395_MOESM8_ESM.tif]

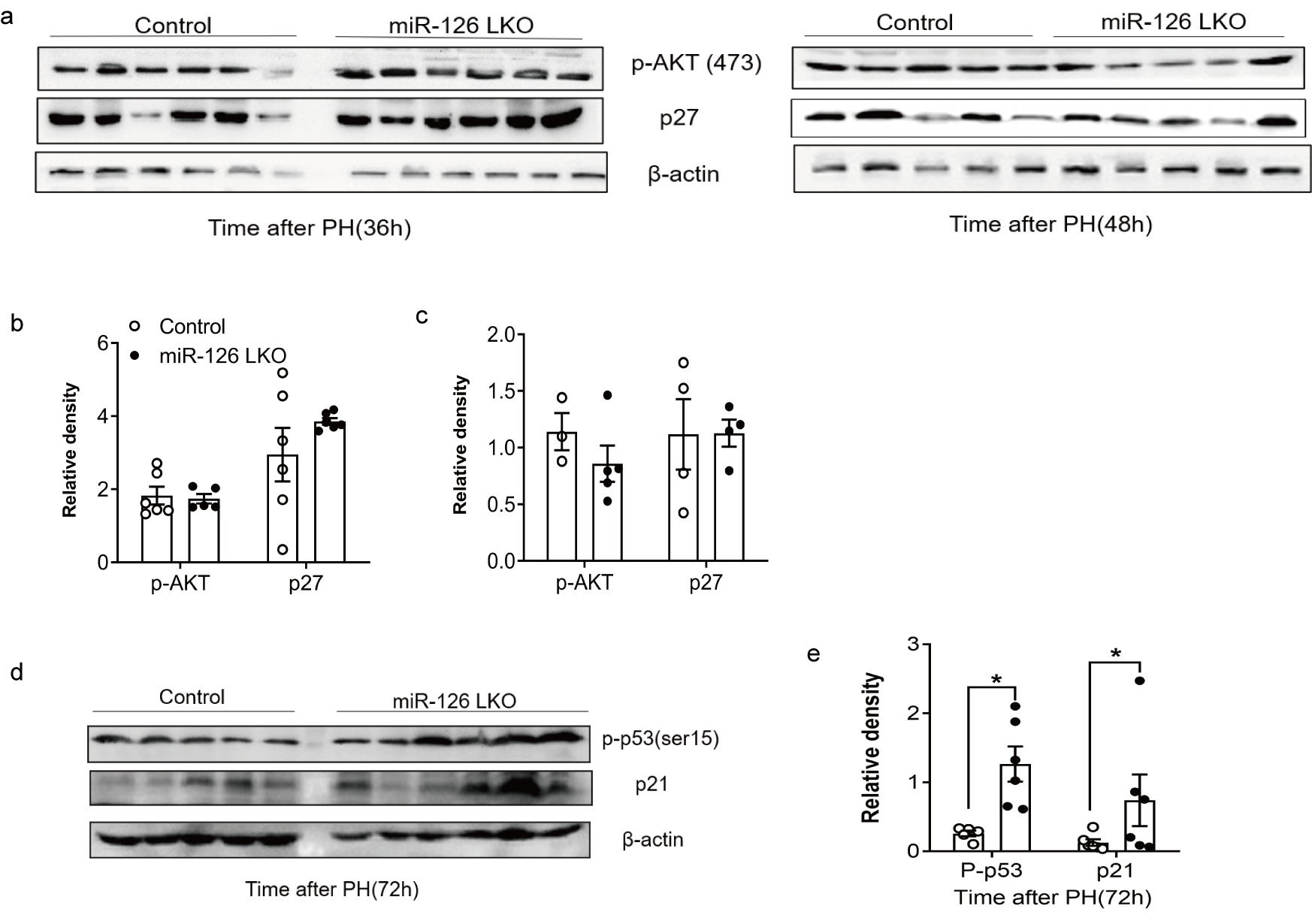

Supplement: Supplementary file 9 — Supple Fig 8a-e [file 41392_2020_395_MOESM9_ESM.tif]

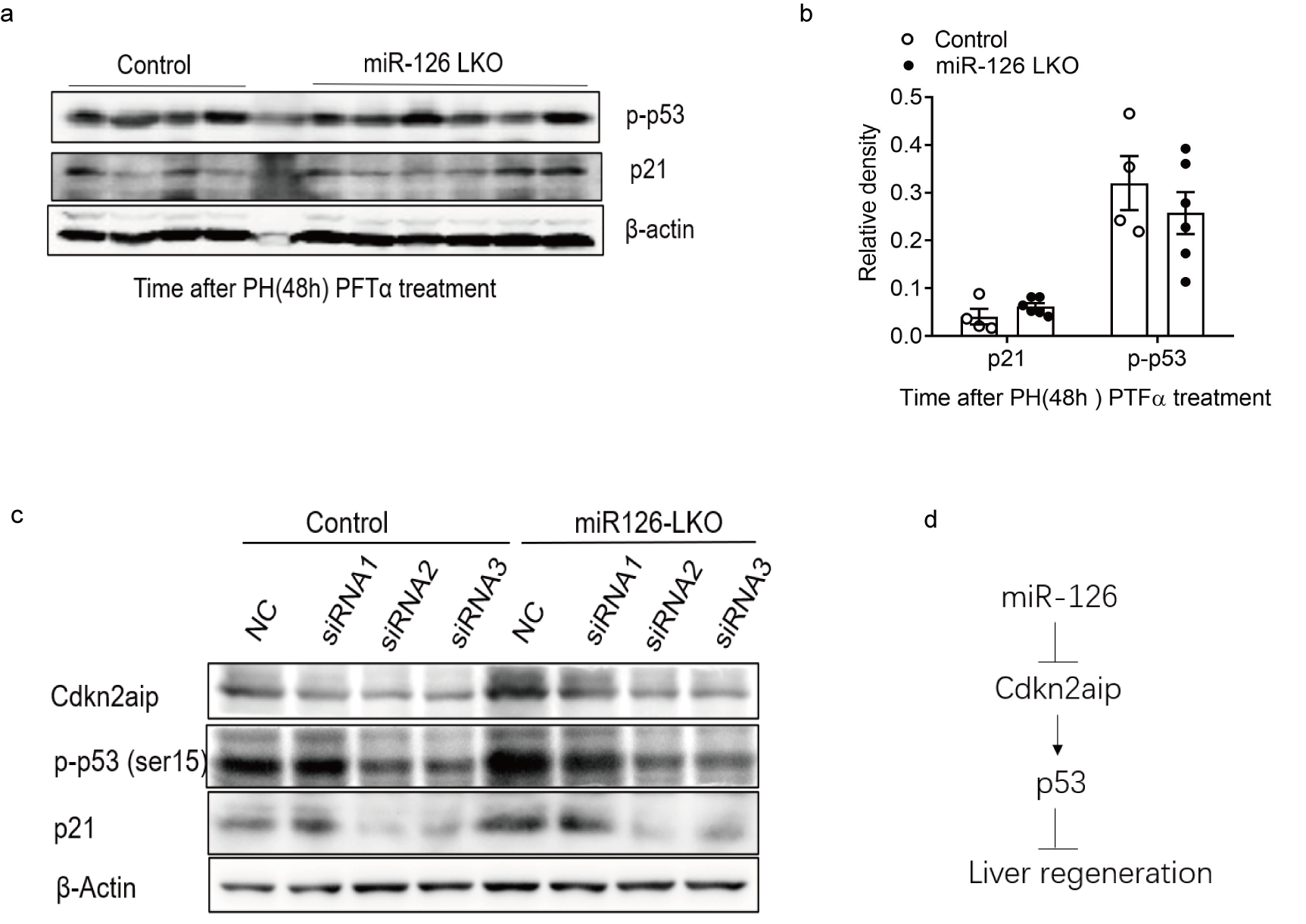

Supplement: Supplementary file 10 — Supple Fig 9a-d [file 41392_2020_395_MOESM10_ESM.tif]
